# Increased oxidative stress and renal injury in patients with sepsis

**DOI:** 10.3164/jcbn.17-130

**Published:** 2018-03-17

**Authors:** Junko Yamaguchi, Midori Nagase, Yorihiro Yamamoto, Atsushi Sakurai, Airi Kubo, Hikaru Mitsuhashi, Masaru Matsuoka, Shingo Ihara, Kosaku Kinoshita

**Affiliations:** 1 Division of Emergency and Critical Care Medicine, Department of Acute Medicine, Nihon University School of Medicine, 30-1 Oyaguchi Kamimachi, Itabashi-ku, Tokyo 173-8610, Japan; 2 School of Bioscience and Biotechnology, Tokyo University of Technology, 1404-1 Katakura-cho, Hachioji, Tokyo 192-0982, Japan

**Keywords:** coenzyme Q10, prosaposin, lecithin-cholesterol acyltransferase, plasma free fatty acids, uric acid

## Abstract

Sepsis remains one of the leading causes of death in intensive care units. The early phase of sepsis is characterized by a massive formation of reactive oxygen and nitrogen species such as superoxide and nitric oxide. However, few comprehensive studies on plasma antioxidants have been reported. Increased oxidative stress was confirmed in sepsis patients (*n* = 18) at the time of hospitalization by a significant decrease in plasma ascorbic acid and a significant increase in the percentage of oxidized form of coenzyme Q10 in total coenzyme Q10 compared to age-matched healthy controls (*n* = 62). Tissue oxidative damage in patients was suggested by a significant decrease in polyunsaturated fatty acid contents and a significant increase in oleic acid contents in total free fatty acids. Thus, it is reasonable that plasma uric acid (end product of purines) would be significantly elevated. However, uric acid levels were continuously decreased during hospitalization for 7 days, indicating a continuous formation of peroxynitrite. A greater decrease in free cholesterol (FC) compared to cholesterol esters (CE) was observed. Thus, the FC/CE ratio significantly increased, suggesting deficiency of lecithin-cholesterol acyltransferase secreted from the liver. Plasma levels of prosaposin, a coenzyme Q10 binding protein, significantly decreased as compared to healthy controls. This may be correlated with renal injury in sepsis patients, since the kidney is thought to be a major secretor of prosaposin.

## Introduction

Sepsis, defined as a “life-threatening organ dysfunction caused by a dysregulated host response to infection”,^(1)^ remains one of the leading causes of death in intensive care units (ICU).^(2–4)^ Since sepsis is caused by a viral, fungal or bacterial infection, the early phase of sepsis is characterized by a massive formation of reactive oxygen and nitrogen species such as superoxide and nitric oxide.^(5–7)^ Peroxynitrite, which is produced from the combination of superoxide and nitric oxide, is also an important reactive molecule.^(5–7)^

Plasma (serum) levels of endogenous antioxidants such as vitamin E (VE), ubiquinol-10 (reduced form of coenzyme Q10), vitamin C (VC), and uric acid (UA) are good scavengers of reactive oxygen and nitrogen species and are thus likely reduced in patients with sepsis; however, no comprehensive studies have been reported. Instead, high serum UA levels are considered to be a marker of sepsis severity.^(8,9)^ In this study, we measured plasma levels of the above antioxidants in patients with sepsis in comparison to age-matched healthy controls.^(10)^ We focused on the percentage of oxidized form of coenzyme Q10 in total coenzyme Q10 because oxidative stress is defined as a disturbance in the prooxidant-antioxidant balance in favor of the former^(11)^ and plasma ubiquinol-10 is vulnerable to oxidation.^(12,13)^

We also employed plasma free fatty acids (FFA) and the content of oxidatively vulnerable polyunsaturated fatty acids in total FFA (%PUFA) as markers of tissue oxidative damage.^(14)^ Stearoyl-CoA desaturase is commonly activated to compensate for the loss of PUFA; therefore, the percentages of palmitoleic acid and oleic acid in total FFA (%16:1 and %18:1, respectively) are also appropriate markers of tissue oxidative damage.^(14)^

Moreover, the ratio of plasma free cholesterol (FC) to cholesterol esters (CE) is of interest since the ratio is determined by the activity of lecithin-cholesterol acyltransferase (LCAT),^(15,16)^ which converts FC to CE. LCAT is secreted with HDL from the liver, and a high FC/CE ratio indicates some impairment of liver function.^(15,16)^

Finally, we measured time course changes of the above antioxidants and lipids during the ICU stay in order to clarify the point at which oxidative stress and tissue oxidative damage become apparent. We suggest that these finding would be useful to decrease the mortality of sepsis patients in the ICU.

## Subjects and Methods

### Study design

The study protocols were approved by the Clinical Research Review Committee of Nihon University School of Medicine (RK-1407115, RK-1509083) and the study was designed as a prospective observational investigation. Patient samples were obtained in accordance with the Helsinki Declaration of 1964, as revised in 2001.

The inclusion criteria were: adult patients with diagnosis of severe sepsis or septic shock according to the International Sepsis Definitions Conference consensus criteria.^(17)^ Exclusion criteria were: age of less than 20 years and patients who received parental or enteral nutrition before hospitalization. Patients who were admitted to the ICU of our hospital between July 2012 and March 2015 and who were diagnosed with sepsis were targeted.

Eighteen adult patients (8 males and 10 females; mean age 74.8 ± 15.1 years) were enrolled after they had given informed consent to participate in the study. Four patients died at 2, 5, 17 and 24 days after admission. There were no significant differences in age and gender between the sepsis patients and healthy volunteers. Body mass index (BMI) was 21.1 ± 5.03 in the sepsis group. Origins of sepsis included pulmonary (*n* = 9), urogenital (*n* = 3), abdominal (*n* = 3), orthopedic (*n* = 1), and various others (*n* = 2).

Heparinized plasma was collected when patients were hospitalized and at 6 and 24 h, and 2 and 7 days. Plasma samples were stored at −80°C until analysis.

The recorded patient information and laboratory data included: age, gender, and sequential organ failure assessment (SOFA) score. BMI was used as an indicator of the general nutritional condition of the patient, using the following formula: body weight divided by the square of body height. Patients who received parenteral or enteral nutrition before hospitalization were excluded from the study because of the possibility that this may have an influence on lipid metabolism. The outcome was evaluated at 28 days in ICU or when the patient was discharged or transferred from our hospital.

SOFA score value for total bilirubin in sepsis patients was 1.4 ± 1.1 (mg/dl), 1.1 ± 1.0, 1.1 ± 1.0 and 0.9 ± 0.6 respectively at 0, 24, 48 and 168 h. There were no patients with severe liver failure, except for one with liver cirrhosis showing 3.46 mg/dl of total bilirubin. However, the liver function of this patient improved at 168 h to 1.94 mg/dl of total bilirubin.

### Analytical procedures

Plasma levels of VE, ubiquinol-10, ubiquinone-10, FC, and CE were determined as previously described with some modifications.^(18)^ In brief, plasma was extracted with 19 volumes of 2-propanol and the extract was analyzed by HPLC using an analytical column (Supelcosil ABZ+, 3 µm, 3.3 cm × 4.6 mm i.d. and Ascentis LC-8, 5 µm, 25 cm × 4.6 mm i.d.; Supelco Japan, Tokyo, Japan) connected in tandem, a reduction column (RC-10-1; Irica, Kyoto, Japan) and an amperometric electrochemical detector (Model Σ985; Irica) with an oxidation potential of +600 mV (vs Ag/AgCl) on a glass carbon electrode. The mobile phase consisted of 50 mM sodium perchlorate in methanol/2-propanol (9/1, v/v), delivered at a flow rate of 0.8 ml/min. The analytical columns were cooled to 25°C.

Plasma levels of VC, UA and unconjugated bilirubin (BR) were determined by HPLC on a bonded-phase aminopropylsilyl column (Supelcosil LC-NH_2_, 5 µm, 25 cm × 4.6 mm i.d.; Supelco Japan) with UV/VIS detection (265 nm for 0–15 min and 460 nm for 15–22 min) as described previously.^(19)^

Plasma FFA were derivatized with monodansylcadaverine for analysis by HPLC.^(20)^ Briefly, plasma samples (50 µl) were mixed with 200 µl of methanol and then centrifuged at 13,000 × *g* for 5 min. Aliquots (50 µl) of supernatants were mixed with 20 µl of methanol containing 25 µM tridecanoic acid (internal standard) and dried under a stream of nitrogen gas, and the residue was admixed with diethyl phosphorocyanidate (1 µl) and *N*,*N*-dimethylformamide (50 µl) containing monodansylcadaverine (2 mg/ml) and kept at room temperature in the dark for 20 min. A 5-µl sample was injected onto an octadecylsilyl column (3 µm, 3.3 cm × 4.6 mm i.d.; Supelco Japan) and a pKb-100 column (5 µm, 25 cm × 4.6 mm i.d.; Supelco Japan) connected in tandem. The FFA components were measured by fluorescence detection (Model 821-FP; Japan Spectroscopic, Tokyo, Japan) with excitation at 320 nm and emission at 520 nm. The mobile phase consisted of acetonitrile/methanol/water (17.5/65.0/17.5, v/v/v) delivered at a flow rate of 1.5 ml/min. The analytical columns were heated to 40°C.

Plasma levels of prosaposin (Psap), a coenzyme Q10 binding and transfer protein, were measured by a sandwich ELISA using monoclonal and polyclonal antibodies against human saposin B.^(21)^ Plasma was diluted 100 times with phosphate-buffered saline containing 0.1% Triton X-100, 1 g/L NaN_3_, 10 g/L BSA, and 1 mM EDTA. Purified saposin B was used as a standard.^(21)^

### Statistical analysis

Data presented are mean values and standard deviations. Statistical analysis was performed with Mann-Whitney’s *U* test for two comparisons and one-way repeated measures ANOVA followed by Scheffe’s multiple comparisons test. *P*<0.05 was considered statistically significant.

## Results and Discussion

### Oxidative stress in sepsis patients

Table 1 shows plasma levels of antioxidants and lipids in sepsis patients at the time of hospitalization and corresponding serum levels in age- and gender-matched healthy controls. A significant decrease in VC levels was observed in sepsis patients compared to healthy controls. No significant differences were observed in levels of BR, VE and total coenzyme Q10 (TQ10). However, a significant increase in the percentage of oxidized form of coenzyme Q10 (%CoQ10) in TQ10 was observed in sepsis patients compared to healthy controls, indicating that the redox balance of coenzyme Q10 shifted to the oxidized form, and confirming the increase in oxidative stress in the blood of sepsis patients. The above results are consistent with reports that VC and ubiquinol-10 are the front-line antioxidants against oxidative stress.^(12,13)^ Elevation of %CoQ10 was confirmed in patients with hepatitis, cirrhosis, hepatoma,^(16)^ Parkinson’s disease,^(22)^ juvenile fibromyalgia,^(23)^ amyotrophic lateral sclerosis (ALS),^(24)^ and post-cardiac arrest syndrome,^(25)^ and in newborn babies^(26)^ and centenarians.^(10)^

### Tissue oxidative damage and plasma UA levels in sepsis patients

Plasma levels of FFA in sepsis patients were higher than in healthy controls, but not significantly so. A significant decrease in %PUFA and a significant increase in %18:1 were observed in sepsis patients as compared to healthy controls (Table 1). These results indicate obvious tissue oxidative damage in sepsis patients. A significant increase in FFA levels and %18:1, and a significant decrease in %PUFA were observed in patients with hepatitis, cirrhosis, hepatoma,^(16)^ juvenile fibromyalgia^(23)^ and post-cardiac arrest syndrome.^(25)^

Tissue damage results in the decomposition of DNA and the conversion of purines to UA. This is consistent with the observed significant increase in plasma UA (Table 1). It is therefore reasonable that the acute physiology and chronic health evaluation (APACHE) II score increases with increasing plasma UA levels.^(8,9)^

### Plasma cholesterol levels and liver function

 Table 1 shows that plasma levels of FC, CE and total cholesterol (TC = FC + CE) were significantly decreased in sepsis patients as compared to healthy controls. Since the decrease of CE levels from healthy control values was more profound than that of FC levels, the FC/CE ratio was greater in sepsis patients compared to healthy controls, although not significantly so. Since the FC/CE ratio is determined by LCAT activity, which converts FC to CE, and LCAT is secreted with HDL from the liver, a high FC/CE ratio indicates some impairment of liver function. A significant increase in the ratio of FC/CE was observed in juvenile fibromyalgia,^(23)^ ALS,^(24)^ and post-cardiac arrest syndrome.^(25)^

### Time course changes

**Fig. 1:**
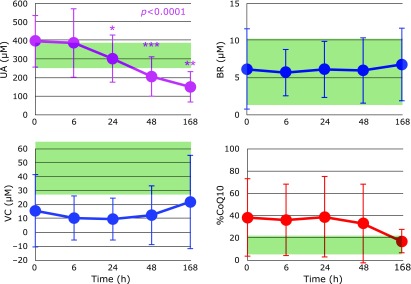
Time course of changes in plasma uric acid (UA), ascorbic acid (VC), unconjugated bilirubin (BR), and the percentage of oxidized form of coenzyme Q10 in total coenzyme Q10 (%CoQ10) after hospitalization. Range of average values ± SD in age-matched healthy controls is shaded in green. *P* values are indicated when one-way repeated measures ANOVA analysis was significant. *p<0.05, **p<0.01 and ***p<0.001, significant differences compared to values at 0 h as determined by Scheffe’s multiple comparison test.

Figure 1 shows the time course of changes in plasma UA, VC, BR, and %CoQ10 after hospitalization. It is obvious that UA levels were continuously decreased during 7 days at the ICU, while VC levels tended to increase and %CoQ10 tended to decrease after day 2. BR levels remained unchanged in the normal range. Since UA is a good scavenger of peroxynitrite and VC and ubiquinol-10 are not, the formation of peroxynitrite was suggested to be ongoing until at least day 7. Therefore, a medication targeting peroxynitrite would improve the outcome of sepsis patients. Further studies are required to confirm this hypothesis.

**Fig. 2:**
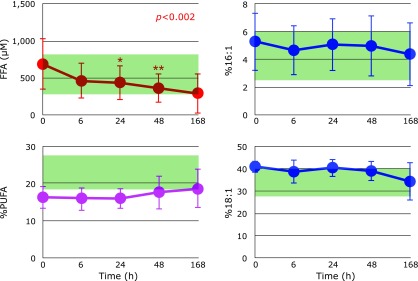
Time course of changes in plasma free fatty acids (FFA), the percentage of polyunsaturated fatty acids in total FFA (%PUFA), the percentage of palmitoleic acid in total FFA (%16:1), and the percentage of oleic acid in total FFA (%18:1) after hospitalization. Range of average values ± SD in age-matched healthy controls is shaded in green. *P* values are indicated when one-way repeated measures ANOVA analysis was significant. *p<0.05 and **p<0.01, significant differences compared to values at 0 h as determined by Scheffe’s multiple comparison test.

Figure 2 shows the time course of changes in plasma FFA, %PUFA, %16:1, and %18:1 after hospitalization. FFA levels decreased continuously and significantly. %PUFA increased to normal levels. Although %16:1 levels remained unchanged in the normal range, %18:1 decreased to normal levels. These results indicate that tissue oxidative damage substantially ceased after day 2.

**Fig. 3:**
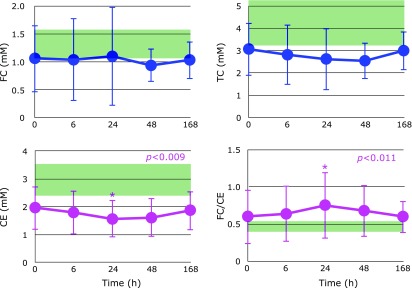
Time course of changes in plasma free cholesterol (FC), cholesterol esters (CE), total cholesterol (TC), and the FC/CE ratio after hospitalization. Range of average values ± SD in age-matched healthy controls is shaded in green. *P* values are indicated when one-way repeated measures ANOVA analysis was significant. *p<0.05, significant differences compared to values at 0 h as determined by Scheffe’s multiple comparison test.

**Fig. 4:**
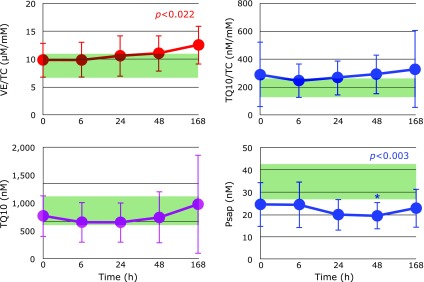
Time course of changes in plasma ratio of vitamin E (VE) to total cholesterol (TC), plasma levels of total coenzyme Q10, ratio of TQ10/TC, and plasma levels of prosaposin (Psap) after hospitalization. Range of average values ± SD in age-matched healthy controls is shaded in green. *P* values are indicated when one-way repeated measures ANOVA analysis was significant. *p<0.05, significant differences compared to values at 0 h as determined by Scheffe’s multiple comparison test.

Figure 3 shows the time course of changes in plasma FC, CE, TC, and the FC/CE ratio after hospitalization. FC levels were unchanged. However, CE levels decreased until day 1 and increased thereafter. Therefore, the FC/CE ratio reached maximum at day 1 then decreased, suggesting that liver function improved after day 1. Figure 4 shows the time course of changes in plasma ratios of VE/TC, TQ10/TC and plasma TQ10 levels after hospitalization. Levels of VE and TQ10 were lowest at 6 h and gradually increased thereafter.

### Oxidative stress, Psap, and kidney function

Psap is the coenzyme Q10 binding and transfer protein present in both the extracellular and intracellular space.^(27)^ We have observed an increase in plasma (serum) levels of Psap under oxidative stress conditions such as in centenarians^(10)^ and patients with post-cardiac arrest syndrome,^(25)^ ALS (unpublished observation), and Parkinson’s disease (unpublished observation). Therefore, it was unexpected that Psap levels in patients with sepsis were significantly lower than those in healthy controls (Table 1). Figure 4 shows that Psap levels were further decreased with time and reached a minimum at day 2. These data indicate that sepsis patients have impairment in the secretion of Psap.

**Fig. 5:**
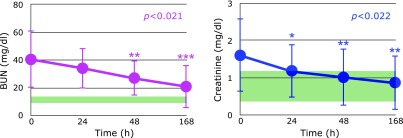
Time course of changes in blood urea nitrogen (BUN) levels and plasma levels of creatinine after hospitalization. Normal range is shaded in green. *P* values are indicated when one-way repeated measures ANOVA analysis was significant. *p<0.05, **p<0.01 and ***p<0.001, significant differences compared to values at 0 h as determined by Scheffe’s multiple comparison test.

**Fig. 6:**
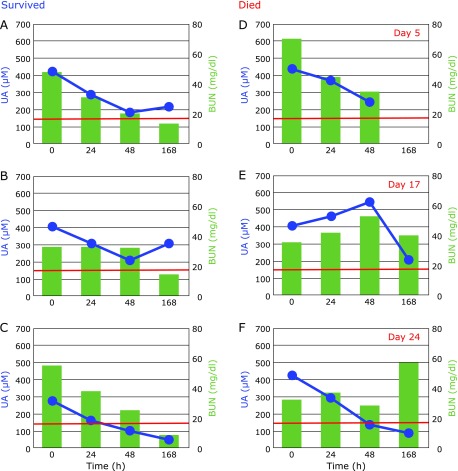
Typical time course of changes in plasma levels of uric acid (UA) and blood urea nitrogen (BUN) after hospitalization. Patients A–C survived and patients D–F died at days 5, 17, and 24. Red lines show the upper limit of BUN normal range.

The kidney is a major secretor of Psap^(27)^ and sepsis induces renal injury^(28)^. In fact, markers of kidney dysfunction, i.e., blood urea nitrogen (BUN) and plasma levels of creatinine, were apparently higher than the normal range, as shown in Fig. 5. These decreased with time, however the patients who died exhibited prolonged elevation of BUN (Fig. 6) and creatinine (data not shown) levels. These results are consistent with the observation that acute kidney injury is a frequent complication of sepsis, and which increases mortality as high as 70%.^(29)^ Therefore, kidney function must be protected in order to decrease mortality in patients with sepsis. The fact that UA continuously decreased indicates the formation of peroxynitrite was not blocked. Thus, administration of edaravone, which is a good scavenger of peroxynitrite, might be beneficial.^(24,30)^ Notably, plasma UA levels increased at day 7 in surviving patients (Fig. 6A and B).

## Conclusion

The increase of oxidative stress in sepsis patients was confirmed by a significant decrease in plasma VC and a significant increase in %COQ10 compared to age-matched healthy controls. UA levels continuously decreased during 7 days of hospitalization, indicating continuous peroxynitrite formation. Plasma levels of Psap, a coenzyme Q10 binding protein, were significantly decreased as compared to healthy controls. This may be correlated with renal injury in sepsis patients, as the kidney is known to be a major secretor of prosaposin.

## Figures and Tables

**Table 1:** Levels of plasma antioxidants, lipids, and prosaposin in sepsis patients at the time of hospitalization as compared to age-matched healthy controls (average ± SD)

	Sepsis	Normal control	*p*
*n*	18	62	
Male/Female	8/10	25/37	
Age (years)	74.8 ± 15.1	75.6 ± 8.1	
VC (µM)	15.1 ± 25.7	46.2 ± 18.4	<0.001
UA (µM)	407 ± 143	327 ± 63	<0.05
BR (µM)	5.9 ± 5.4	6.0 ± 4.6	
VE (µM)	28.1 ± 10.0	33.3 ± 9.8	
TQ10 (µM)	758 ± 368	880 ± 313	
%CoQ10	41.4 ± 36.8	12.7 ± 9.5	<0.05
FFA (µM)	667 ± 359	501 ± 233	
%PUFA	16.0 ± 3.5	23.4 ± 4.9	<0.001
%16:1	5.3 ± 2.0	4.2 ± 1.8	
%18:1	40.9 ± 2.7	33.6 ± 6.6	<0.001
FC (mM)	1.05 ± 0.59	1.31 ± 0.28	<0.001
CE (mM)	1.87 ± 0.87	3.03 ± 0.61	<0.001
TC (mM)	2.96 ± 1.26	4.33 ± 0.83	<0.001
FC/CE	0.74 ± 0.67	0.44 ± 0.08	
10^3^ VE/TC	10.3 ± 3.7	8.6 ± 2.4	<0.05
10^6^ TQ10/TC	303 ± 237	205 ± 69	
Psap (nM)	25.3 ± 10.2	34.8 ± 7.4	<0.01

*P* values were determined using Mann-Whitney’s *U* test. VC, ascorbic acid; UA, uric acid; BR, unconjugated bilirubin; VE, vitamin E; TQ10, total coenzyme Q10; %CoQ10, ratio of oxidized form of coenzyme Q10 to TQ10; FFA, free fatty acids; %PUFA, ratio of polyunsaturated fatty acids to total FFA; %16:1, ratio of palmitoleic acid to total FFA; %18:1, ratio of oleic acid to total FFA; FC, free cholesterol; CE, cholesterol esters; TC, total cholesterol; Psap, prosaposin.

## Abbreviations

%16:1: percentage of palmitoleic acid in total FFA
%18:1: percentage of oleic acid in total FFA
BR: unconjugated bilirubin
BUN: blood urea nitrogen
CE: cholesterol esters
%CoQ10: percentage of oxidized form of coenzyme Q10 in TQ10
FC: free cholesterol
FFA: free fatty acids
LCAT: lecithin-cholesterol acyltransferase
Psap: prosaposin
%PUFA: percentage of polyunsaturated fatty acids in total FFA
SOFA: sequential organ failure assessment
TC: total cholesterol
TQ10: total coenzyme Q10
UA: uric acid
VC: ascorbic acid
